# Meningococcal B Vaccines as a Paradigm of Safe and Effective Vaccines for Children

**DOI:** 10.3390/vaccines13070770

**Published:** 2025-07-21

**Authors:** Maribel Gonzalez Tome, Rosa Gonzalez-Quevedo, Maria Escudeiro dos Santos, Hans Juergen Dornbusch, Sabine Straus, Emer Cooke

**Affiliations:** 1 Vaccines and Therapies for Infectious Diseases, Human Medicines Division, European Medicines Agency, Domenico Scarlattilaan 6, 1083 HS Amsterdam, The Netherlands; 2 Public and Stakeholder Engagement Department, European Medicines Agency, Domenico Scarlattilaan 6, 1083 HS Amsterdam, The Netherlands; 3 European Academy of Paediatrics (EAP)/Union of European Medical Specialists—Section of Paediatrics (UEMS-SP), Avenue de la Couronne, 20, B-1050 Brussels, Belgium; 4 Institute of Hygiene, Microbiology and Environmental Medicine, Medical University of Graz, 8036 Graz, Austria; 5 Pharmacovigilance and Risk Assessment Committee (PRAC), European Medicines Agency, Domenico Scarlattilaan 6, 1083 HS Amsterdam, The Netherlands; 6 European Medicines Agency, Domenico Scarlattilaan 6, 1083 HS Amsterdam, The Netherlands

**Keywords:** invasive meningococcal disease, *Neisseria meningitidis* serogroup B, vaccine, regulatory framework, effectiveness, safety monitoring system

## Abstract

Background: *Neisseria meningitidis* B is one of the main causative pathogens of meningitis and other forms of severe meningococcal disease. In the past decade, meningococcal B vaccines have been developed to address this infection and its sequelae. Objective: This article aims to present an example of how the EU regulatory framework allowed the early authorisation of two life-saving vaccines initially based on immunogenicity surrogates of clinical evidence. This was subsequently followed by post-marketing surveillance providing real-world evidence to support their safety profile and impact on the paediatric population in the EU. Methods: We review the evidence supporting the initial regulatory approval of the vaccines, the confirmatory data demonstrating vaccine effectiveness post-authorisation, and the real-world impact of these vaccines on the paediatric population. Results: Two vaccines were approved in the EU for active immunisation to prevent IMD caused by MenB (4CMenB in 2013 and MenB-fHBP in 2017). Both marketing authorisations were based on immunogenicity data (efficacy studies were not feasible due to the rarity of the disease) and safety data generated from pre-authorisation studies. Additional pharmacovigilance activities to further investigate the safety profile and effectiveness studies were requested to be conducted after approval. Both the effectiveness and safety profile of the vaccines were confirmed by these data. Conclusions: This paper illustrates that the EU medicines regulatory framework and safety monitoring system are robust. By supplementing the initial evidence with post-authorisation studies, further effectiveness and safety data enabled regulators to confirm the positive benefit–risk of the vaccines without delaying their access to the people who need them.

## 1. Introduction

Invasive meningococcal disease (IMD), including meningococcal B infection, still poses diagnostic challenges. In addition, despite the availability of antibiotic treatment, around 1 in 10 people with IMD die, and up to a quarter develop significant sequelae, such as loss of limbs, epilepsy, or learning difficulties [1,2,3]. Meningococci belonging to six serogroups (A, B, C, W, X, and Y) cause nearly all IMD worldwide, with serogroup B meningococci (MenB) being the predominant cause in many European countries [4]. IMD is a major public health problem and remains a leading cause of meningitis and sepsis in many countries. Its high fatality rate and the seriousness of the sequelae prompted patient groups and clinicians to request vaccines for this unmet medical need.

**Figure 1:**
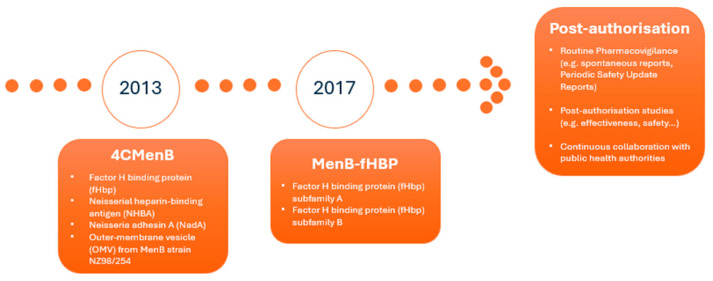
Timeline for approval, vaccine composition, and post-authorisation monitoring.

Two companies responded by developing vaccines targeting MenB and submitting marketing authorisation applications to EMA for evaluation via the centralised procedure. This led to the approval of two vaccines in the EU: 4CMenB (Bexsero^®^) was approved in 2013 for the active immunisation of individuals from 2 months of age and older against IMD caused by *N. meningitidis* serogroup B, [5] and MenB-fHBP (Trumenba^®^) was authorised in 2017 for the active immunisation of individuals 10 years and older to prevent IMD caused by the same pathogen [6] [Figure 1].

Regarding the antigen composition, 4CMenB consists of three recombinant proteins, namely factor H binding protein (fHbp), Neisserial heparin-binding antigen (NHBA), and Neisseria adhesin A (NadA). These were originally identified by a reverse vaccinology approach, combined with the outer-membrane vesicle (OMV) from MenB strain NZ98/254, which contains porin A (PorA) serosubtype P1.4. The MenB-fHBP vaccine contains two fHbp antigen subvariants: peptide 45 and peptide 55 [Figure 1]. 

At the time of the initial marketing authorisation application, no efficacy data were available, since meningococcal efficacy trials in non-epidemic settings are not feasible due to the rarity of the disease [7,8]. This required the EU regulatory framework to support earlier access to these critical vaccines by evaluating their risks based on clinical evidence of safety and their benefits based on vaccine immunogenicity. Such immunogenicity was studied by measuring the ability of the sera from immunized persons to kill the bacteria in vitro in a serum bactericidal assay (SBA) [9], which had been established as a correlate for protection for meningococcus C already in 1969, with the understanding that proof of effectiveness would only be achievable in the context of post-authorisation studies in much larger populations.

This paper thus presents an example of how the EU regulatory framework allowed the early authorisation of two life-saving vaccines based on promising clinical evidence and how robust post-marketing surveillance subsequently provided real-world evidence of their safety and impact on the paediatric population in the EU.

## 2. Methods

In this article, a review of the evidence supporting the initial regulatory approval of the vaccines, the confirmatory data demonstrating vaccine effectiveness post-authorisation, and the real-world impact of these vaccines on the paediatric population is presented. For this, a review of the procedures submitted to EMA related to MenB vaccines, including their initial submission to apply for marketing authorisation and the post-authorisation procedures, was considered [Figure 1]. In addition, a review of the literature was performed. Finally, the regulators’ and paediatricians’ perspectives were added, the latter provided by the European Academy of Paediatrics.

## 3. Results

### 3.1. Data Supporting Regulatory Approval

#### 3.1.1. Efficacy–Immunogenicity

Both marketing authorisation applications were based on immunogenicity data using a serological surrogate marker for protection. The SBA measures a functional immune response to the vaccine, which results in the recognition of bacterial surface antigens and subsequent complement-mediated bacterial lysis, thereby mimicking the main mechanism by which *N. meningitidis* serogroup B strains are killed after natural infection. The assay can give information on whether a particular serum sample has a level (titre) of bactericidal antibodies sufficient to reach a protective threshold. Robust primary SBA responses were demonstrated in the relevant age groups against the main antigens for different MenB strains [7,8].

#### 3.1.2. Safety

For 4CMenB, safety was assessed pre-authorisation in 8 studies comprising over 6400 individuals from 2 months to 50 years of age, with age-specific dose schedules. Most adverse events (AEs) observed after each vaccination were mild (few were moderate); local reactions (pain or tenderness, swelling, hardness, and redness of the skin at the injection site), headache, and arthralgia were the most commonly reported AEs across age groups. In infants and children up to 10 years of age, eating disorders, sleepiness, unusual crying, diarrhoea, vomiting, rash, fever, and irritability were also commonly reported. In adults and adolescents from 11 years of age, nausea, malaise, and myalgia were common side effects. When infants were given 4CMenB alone, post-vaccination fever ≥ 38 °C was seen at frequencies comparable to those observed in clinical trials with other routine infant vaccines. However, when the vaccine was co-administered with routine vaccines, fever was more common compared with vaccination with 4CMenB alone [5].

The safety of MenB-fHBP was investigated in 11 pre-authorisation clinical studies in over 15,000 participants aged 10 years and older. Most AEs observed after vaccination were mild to moderate; these included local reactions (pain, redness, and swelling at the injection site), headache, fatigue, chills, diarrhoea, muscle or joint pain, and nausea. Fever ≥ 38 °C was common in clinical studies [6].

For both vaccines, based on the data generated from studies at the time of marketing authorisation, the most commonly observed adverse reactions were injection site reactions (such as pain and swelling), fever, and fatigue. The safety profile was considered comparable to other vaccines routinely administered in the related age groups [7,8].

Companies are required to submit a risk management plan (RMP) for medicines’ regulatory approval in the EU. The RMP is a standard document that details any important risks of a medicine (identified or potential) and how such risks can be minimised. It also includes the studies (or specific obligations) to be conducted post-marketing by the companies to further characterise risks or remaining uncertainties about the medicine’s safety.

For both vaccines, EMA’s Pharmacovigilance Risk Assessment Committee (PRAC) requested additional pharmacovigilance activities in addition to routine pharmacovigilance to further investigate the safety concerns identified at the time of approval. These additional pharmacovigilance activities were included in the pharmacovigilance plan of the RMP, which presents an overview and discussion on how the marketing authorisation holder (MAH) plans to further characterise the safety concerns [10,11,12] [Figure 1].

### 3.2. Data Generated Post-Approval

#### 3.2.1. Effectiveness

As part of the terms of the marketing authorisation, the MAHs were requested to carry out effectiveness studies after approval, in collaboration with public health authorities leading the vaccination campaigns.

For 4CMenB, an observational study (V72_38OB) was conducted by Public Health England (PHE) in the context of the national infant immunisation programme (NIP) in the UK. The aim was to generate evidence on vaccine effectiveness (VE), which indicates the protection conferred by immunisation in a vaccinated population compared with unvaccinated controls once vaccines are used in a real-world setting, outside the controlled setting of clinical trials. The study also analysed vaccine impact (VI) on infants, defined as the reduction in IMD cases in children once the vaccine was introduced, compared with a previous period when no vaccine was available.

The 4CMenB vaccine was administered as a 2-dose schedule at 2 and 4 months of age, followed by a booster dose at 12–13 months of age. The surveillance program monitored vaccine uptake in the eligible birth cohorts, IMD cases, and VE and VI in the population under 5 years of age. In addition, vaccination failures (VFs) were also measured to analyse the potential lack of efficacy. VF was evaluated using the meningococcal antigen typing system (MATS), previously used to determine which portion of the existing MenB strains in a country would be covered by the 4CMenB vaccine. The MATS method predicts whether a given MenB isolate would be susceptible to killing in the serum bactericidal assay using human complement (hSBA) by antibodies induced by the vaccine [13]. MATS data allow for the evaluation of VF cases against vaccine-preventable strains.

The final 3-year data showed a statistically significant 75% reduction in IMD cases caused by *N. meningitidis* group B (IRR, 0.25; 95% CI: 0.19–0.36); vaccine impact (VI = (1 − IRR) × 100) was calculated in vaccination-eligible infants, irrespective of the infants’ vaccination status or predicted MenB strain coverage, compared to 4 years pre-vaccination. The calculated vaccine impact translated into the prevention of 277 MenB cases (95% CI: 236–323) since 169 cases were observed and 446 were expected in the UK over 3 years. Only 21 cases of confirmed VF were identified among almost 2 million vaccinated infants and toddlers, proving robust protection against IMD caused by MenB [14].

As indicated, completed MATS data were also evaluated to analyse vaccination failures against vaccine-preventable strains as one of the secondary study outcomes [15,16,17]. Overall, the evaluation of the VF cases did not identify any specific concern.

In the UK setting, the first 4CMenB vaccine dose was administered at 2 instead of 3 months of age, and the schedule was 2 + 1 instead of 3 + 1, as initially recommended in the EU SmPC. As stated in Section 4.1 of the EU’s Summary of Product Characteristics (SmPC) [5], national vaccination authorities can set up their official recommendations on the populations eligible for vaccinations according to local criteria. After millions of infants had received a first dose of 4CMenB at 2 months of age without any efficacy or safety concerns, EMA’s Committee for Medicinal Products for Human Use (CHMP) recommended lowering the age of first vaccination to 2 months, and the EU product information was updated accordingly [5].

Completion of the V72_38OB study and regulatory endorsement of the vaccine’s effectiveness led to the removal of the study from the pharmacovigilance plan, thus formally complementing the immunogenicity data and demonstrating the positive impact of the 4CMenB vaccine.

To evaluate the effectiveness of MenB-fHBP in reducing the incidence of IMD (serogroup B) in the indicated population, the MAH was requested to collaborate with relevant public health authorities and clinical investigators with access to integrated clinical and epidemiological IMD databases in regions where the vaccine is used as part of a national immunisation program or in response to hyperendemic or large outbreak situations.

As real-world data continue to be collected for MenB-fHBP, detailed meningococcal epidemiological reports and effectiveness information are regularly provided in the periodic safety update reports (PSURs). PSURs are pharmacovigilance documents sent by MAHs to EMA for assessment, and they are intended to provide an evaluation of the risk–benefit balance of a medicinal product at defined time points after its authorisation. In these routine safety reports, the MAH also details the progress made towards executing a study to evaluate the effectiveness of the vaccine in the US (where it is mainly distributed), in Europe, and in any country that introduces MenB-fHBP into a national or regional immunisation programme [11].

Clinical development of MenB-fHBP has focused on individuals aged ≥ 10 years. In the medical literature, protective titres against MenB test strains were reported after 2- and 3-dose schedules [18,19,20]. In addition, in individuals who received a single booster dose four years after receipt of a primary series of 2 or 3 doses of the MenB-fHBP vaccine, the booster elicited robust immune responses, as measured by hSBA at 1 month post-booster. The persistence of protective antibody titres for up to four years has also been reported for over 50% of study participants [21,22], and the vaccine has been effective in controlling outbreaks [23].

#### 3.2.2. Safety

Both vaccines demonstrated an acceptable safety profile at approval and, as is the case for all medicines, the risk profile continues to be closely monitored by pharmacovigilance in the real-world setting. This relies on tools such as spontaneous reports [24,25], post-authorisation studies [26], and PSURs.

The main objective of a PSUR is to present a comprehensive, concise, and critical analysis of the risk–benefit balance of the medicinal product, taking into account new or emerging information in the context of cumulative evidence on risks and benefits. The PSUR is therefore a tool for post-authorisation evaluation at defined time points in the lifecycle of a medicine [27].

The assessed PSURs of both vaccines did not raise any significant changes in the benefit–risk profile concerning the approved indication; however, the product information was updated to reflect the most recent data. Currently, both vaccines have a PSUR frequency of 3 years, and the MAHs comply with these reporting frequencies.

Routine pharmacovigilance further allows continuous monitoring of the safety profile and allows the timely identification of adverse reactions (such as allergic reactions for both vaccines) to be added to the product information. They have also allowed updating frequencies of adverse reactions (such as urticaria and headache for 4CMenB) already listed in the product information and expanding the existing information (for example, on the risk of fever for both vaccines) based on the ‘real-life’ use of the vaccines.

In addition to routine measures, post-authorisation safety studies (included as additional pharmacovigilance activities in the RMP) further supported the safety data reviewed in PSURs. The results from post-approval observation studies confirmed the known safety profile of the vaccines.

## 4. Discussion

Following the regulatory approval of vaccines, national authorities discuss whether they should be introduced in national immunisation programmes. The potential factors to be considered are related to the disease like incidence, serogroup distribution and fatality rate (which varies between countries), the vaccine’s immunogenicity, efficacy, safety, effectiveness and impact including strain coverage, and effect on bacterial carriers (i.e., *N. meningitidis* is a human commensal which commonly colonises the oropharyngeal mucosa), health economic evaluations, population acceptance, etc. [28]. Initially, the use of these vaccines was more limited, but as more post-marketing evidence emerged over the years, it actively contributed to the inclusion of MenB vaccines into more national immunisation schedules. Although not always included in the national immunisation programmes, their use in clinical practice continues to generate real-world data for both MenB vaccines. Both vaccines have been used to manage outbreaks, and both are currently approved in more than 50 countries worldwide [29,30,31].

While IMD incidence declined in 2020 and 2021, recent data from the European Centre for Disease Prevention and Control (ECDC) indicates that in 2022, the notification rate of IMD rose to 0.3 cases per 100,000 population. The notification rate of serogroup B returned to that observed prior to the COVID-19 pandemic or even lower in all age groups except for individuals aged 15–24 years, where it increased beyond that observed in 2018–2019. Serogroup B has remained predominant with 62% of serogroup documented cases, even if its incidence has declined since 2014 [28]. The use of MenB vaccines and the inclusion in the national immunisation programs could in part explain this decrease, although the effect was also observed in age groups not targeted by vaccination or in countries where vaccination was not implemented. As already indicated, in Europe, vaccination policies vary from one country to another, with heterogeneous IMD immunisation programs, even in neighbouring countries, which complicates the evaluation of the vaccination impact due to confounding factors like different schedules and uptake rates [32,33].

With more than three million doses administered globally, the absence of serious post-vaccination adverse effects was confirmed in real life. The most frequently reported adverse events for 4CMenB, collected through the British Yellow Card surveillance system, were local reactions (inflammation, palpable nodule with slow resolution) and fever. An increase in visits to primary care clinics and hospital emergency services was observed as a consequence of post-vaccine fever without an increase in febrile convulsions [14,34].

For MenB-fHBP, evidence from the medical literature presents fever, headache, injection site pain, and fatigue as the most common adverse events, mostly mild to moderate in severity [35]. The datasets for both vaccines are consistent with the information from the clinical trials [19].

When introducing a new vaccine, a key aspect to consider is the potential emergence of new serogroups, or different strains within existing serogroups, that could be able to escape protection conferred by vaccines and thus render them ineffective as the pathogen evolves. Available data on IMD epidemiological trends over time do not indicate that immunisation with either 4CMenB or MenB-fHBP vaccines induced changes in strains or serogroups in nasopharyngeal colonisation worldwide. Nevertheless, as IMD epidemiology can evolve, continuous monitoring and surveillance are important to determine if the composition of meningococcal vaccines would need to be adapted. In this regard, it is important to note that MenB strains exhibit significant genetic diversity, with different epidemiology across different countries and changes over time, with a direct impact on the predicted coverage of the 4-component MenB vaccine (4CMenB). This diversity arises from variations in the distribution of clonal complexes and antigenic profiles, leading to differences in vaccine effectiveness among regions with genetic Meningococcal Antigen Typing System (gMATS)-predicted coverage ranging from 62.7% in Taiwan to 86% in Finland and 91.4% in Lithuania [36]. gMATS was recently developed and utilises genomic data to predict antigen expression and potential vaccine coverage, facilitating large-scale surveillance. However, its predictive accuracy can be limited by the presence of novel or rare antigen variants lacking corresponding phenotypic data, leaving a proportion of isolates being classified as “unpredictable” [37]. Therefore, the genetic diversity and changing epidemiology of MenB strains necessitate continuous monitoring. Tools like gMATS play a crucial role in this process, enabling the assessment of vaccine coverage and guiding public health interventions, including vaccination strategies.

Apart from the results on IMD prevention presented here, other possible benefits of MenB vaccines suggested in the medical literature include their putative, although non-proven, effect on nasopharyngeal carriage and their potential to induce cross protection against other meningococcal strains [38,39]. MenB vaccines are manufactured using proteins found on the surface of MenB which are also present on other bacteria belonging to the *Neisseria* family. It has been suggested and eventually described that 4CMenB vaccine can also offer protection against *N. gonorrhoea* [40,41,42,43]. In fact, 4CMenB will be rolled out in England and Wales as part of a world-first programme to prevent gonorrhoea in individuals at higher risk [44]. In addition, specific vaccines against *N. gonorrhoea* are currently under development [45].

Initially, protein-based MenB vaccines were developed since polysaccharide-based vaccines were poorly immunogenic, with the additional potential of autoimmune effects due to similarity with a human polysaccharide. Moreover, the development of broadly effective MenB vaccines was complicated due to challenges specific to the MenB capsule, since initial MenB vaccines lacked broad protective activity against heterologous MenB strains. However, this issue was finally overcome by the multicomponent 4CMenB and MenB-FHbp vaccines. Since then, the vaccine development landscape has continued to evolve; new pentavalent vaccines are in development, and two are currently approved by the US Food and Drug Administration (FDA) [46,47]. These vaccines include the meningococcal serogroups A, C, W, and Y in addition to serogroup B and, therefore, can simplify the immunisation schedule by lowering the total number of doses needed to provide protection against the five serogroups responsible for nearly all IMD.

Based on the scientific evidence generated over a decade of real-world clinical use, MenB vaccines represent an example of the successful introduction of new vaccines for children to cover an important unmet medical need. From the regulators’ perspective, the EU regulatory framework used for the approval of meningococcal B vaccines has facilitated early access to promising and needed medicines with the required safeguards of quality, safety, and efficacy. In addition, post-approval evidence of the safety of these vaccines has become very solid as the specific requests to companies have been fulfilled, and the missing information has been generated to resolve the uncertainties that remained at the time of initial approval. Finally, the approval and subsequent implementation in national immunisation campaigns, with proven effectiveness, have also demonstrated that the EU regulatory framework and monitoring system are robust and able to evaluate real-world data as these continue to be generated.

In a time when public health is challenged by increasing mis/disinformation and vaccine hesitancy, exacerbated during the COVID-19 pandemic, it is important to highlight the overwhelmingly positive milestones in the history of vaccination [48]. From the perspective of European paediatricians, MenB vaccines represent a success story on the prevention of a very serious disease affecting children. Vaccination is one of the most effective public health interventions against infectious diseases, not only saving millions of lives yearly but importantly preventing the sequelae of infectious diseases in survivors, which in the case of IMD can be as severe, disabling and life-changing as deafness, limb amputations, epilepsy, and cognitive impairment [49].

In this sense, access to vaccination remains a key issue. National immunisation programmes with epidemiologically driven, country-specific recommendations for the different serogroups have been established in more than 25 countries worldwide [50,51,52]. The majority of EU countries issue similar recommendations for the vaccination of infants, young children, and adolescents.

However, there are still issues to be improved. Socially disadvantaged populations have lower vaccination coverage rates and suffer from a disproportionately high burden of disease, combined with poorer access to health services [53,54]. Two recent studies, a case-control study from France and a retrospective correlational study from Spain, show that low socioeconomic status increases the risk of IMD [55,56]. Children from low-income families, especially infants under one year of age, have an increased risk of hospitalisation due to IMD [57]. An online survey of over 600 US households with at least one adolescent aged 16–19 years identified annual household income as the most influential variable in predicting interest in MenB vaccination [28]. Therefore, vaccination equity needs to be tackled to optimise the protection of children.

From the paediatrician’s perspective, parental knowledge about IMD and vaccination options should be increased by distributing information materials ahead of vaccination to enable an informed decision [58,59]. Vaccination education talks by treating physicians should also be encouraged, and there is a need for further training on vaccination among healthcare professionals. Aiming at reduction in the administrative effort for all (including meningococcal) vaccination programmes, the implementation of national electronic immunisation records (EIR) is encouraged, enabling a quick check of vaccination status, uncomplicated ordering logistics and billing of cost free vaccinations, automated reminder systems, easy analysis of vaccine coverage, and scientific evaluations [60].

As a final note, the World Health Organization (WHO) has presented a global road map to defeat all types of bacterial meningitis (as an important form of invasive disease) by 2030 [61,62]. In this regard, vaccines against *N. meningitidis* are expected to play an important role in diminishing epidemics, deaths, and disease sequelae. Every step taken by the regulatory and paediatric communities to inform the public on the positive impact of these vaccines will effectively contribute to avoiding more vaccine-preventable suffering in children.

## 5. Conclusions

Meningococcal B vaccines illustrate the robustness of the EU regulatory framework and monitoring system and its preparedness for approving new vaccines for childhood diseases. The framework supports early access to promising and needed products with the required quality, safety, and efficacy standards, based on immunogenicity data, while allowing for the generation of more evidence post-marketing to complete the missing information and resolve the uncertainties existing at initial approval, both for effectiveness and safety monitoring. By supplementing the initial evidence with post-authorisation studies, companies continue collecting and providing effectiveness and further safety data, which enables regulators to continue characterising the medicines’ safety and efficacy without delaying their access to the population.

The approval of and clinical experience with MenB vaccines represent an excellent example of how the introduction of new vaccines can help to address unmet medical needs and how vaccination can greatly reduce a severe disease burden for the paediatric population.

However, there are issues yet to be improved, such as the inequality reducing vaccination coverage in some groups, misinformation, and vaccination training level of healthcare professionals. The WHO, in collaboration with partners and donors aims to achieve the ambitious goals to defeat meningitis by 2030.
